# Supplementary material for: Breaking barriers in the sensitive and accurate mass determination of large DNA plasmids by mass photometry
Source: Mol Ther Nucleic Acids. 2025 Jul 17;36(3):102632. doi: 10.1016/j.omtn.2025.102632 (PMC12328894; doi:10.1016/j.omtn.2025.102632)
Supplement: Document S1. Figures S1–S5 [file mmc1.pdf]

OMTN, Volume *36*

Supplemental information

Breaking barriers in the sensitive and accurate mass determination of large DNA plasmids by mass photometry

Eduard H.T.M. Ebberink, Evolène Deslignière, Alisa Ruisinger, Markus Nuebel, Marco Thomann, and Albert J.R. Heck

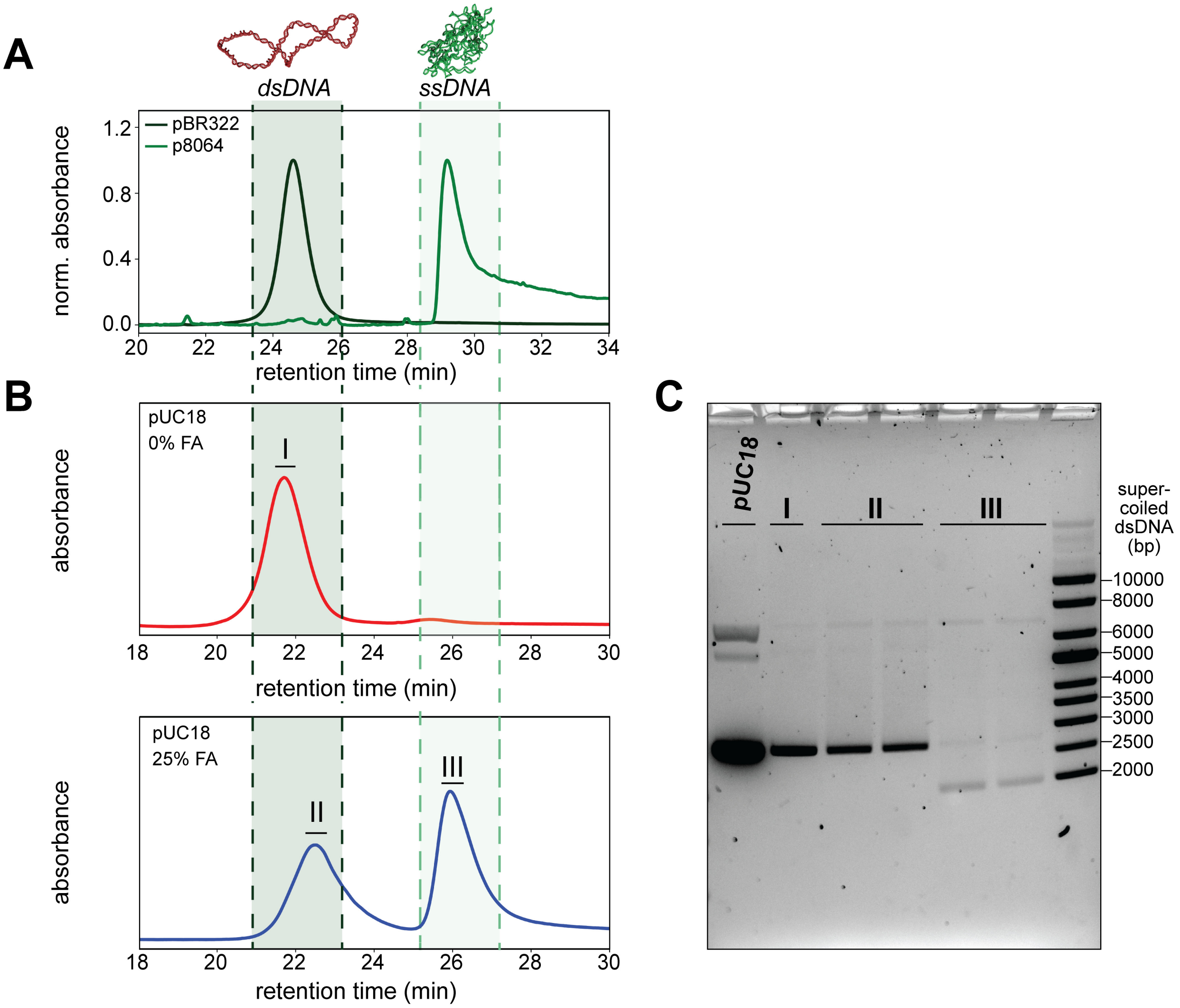

Figure S1: Reversed phase high-pressure liquid chromatography of dsDNA pBR322, pUC18, and ssDNA p8064. A) Overlayed chromatograms of similar sized dsDNA pBR322 and ssDNA p8064 (respectively 2.69 MDa and 2.49 MDa), reveal that these separate very well. Using hydrophobic C18 column as stationary phase, pBR322 elutes at ~25 minutes and p8064 at ~29 minutes. **B)** Before (top) and after (bottom) incubation of pUC18 with formic acid (FA), the dsDNA (I and II) and ssDNA-like particles (III) are clearly separated by HPLC. **C)** Fractions were taken for each peak in panel B and analyzed by gel electrophoresis. In fractions I and II, the DNA behaves as dsDNA, in fraction III it behaves like ssDNA running below the dsDNA band.

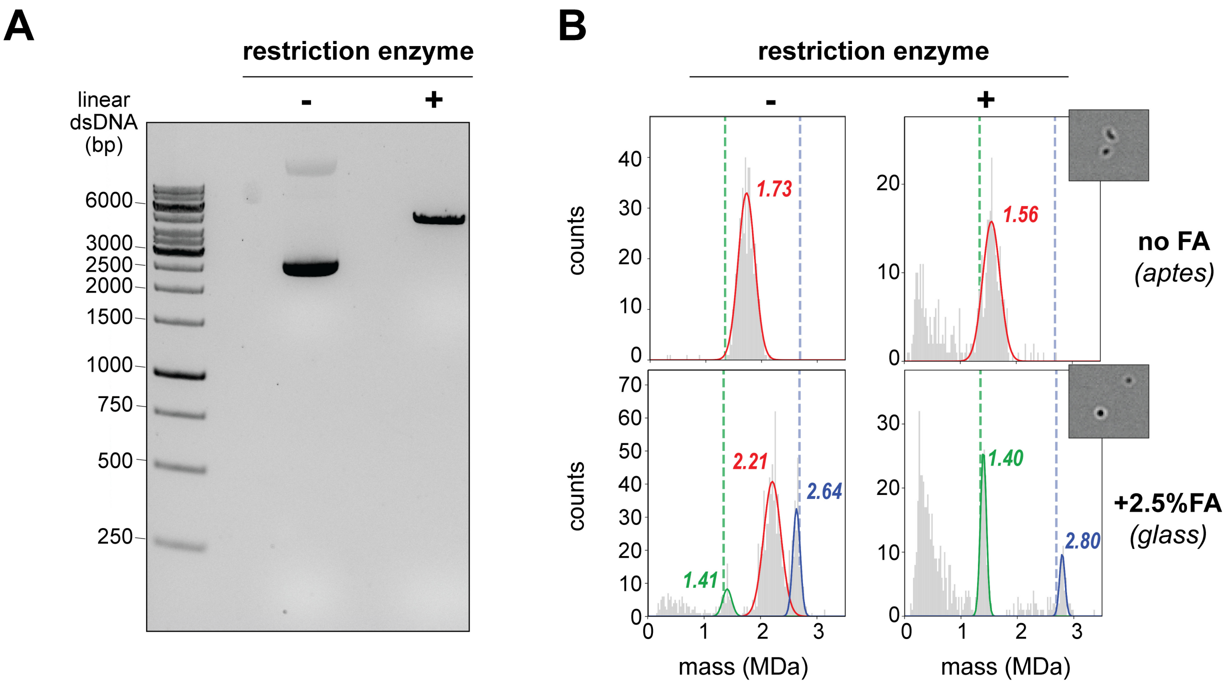

Figure S2: Formic acid-treatment of linearized pBR322. A) By use of a restriction enzyme (Eco32I) the pBR322 was cut once, resulting in a linearized, double-stranded pBR322 of 4361 base pairs. **B)** Representative MP measurements of pBR322 with or without restriction enzyme and with or without 2.5% FA. In the top panels, linearized pBR322 without FA shows similar behavior as the intact supercoiled plasmid with oval-shaped contrast signals and a lower-than-expected mass (inset shows example landing events). In the lower panels, addition of 2.5% FA to linearized pBR322 generates circular landing events, a correct contrast-to-mass conversion and interaction of 1x and 2x ssDNA-like particles with the glass coverslip. The 0.5x and 1x pBR322 mass is indicated by respectively green and blue vertical, dashed lines. The average masses of the detected populations are given in MDa.

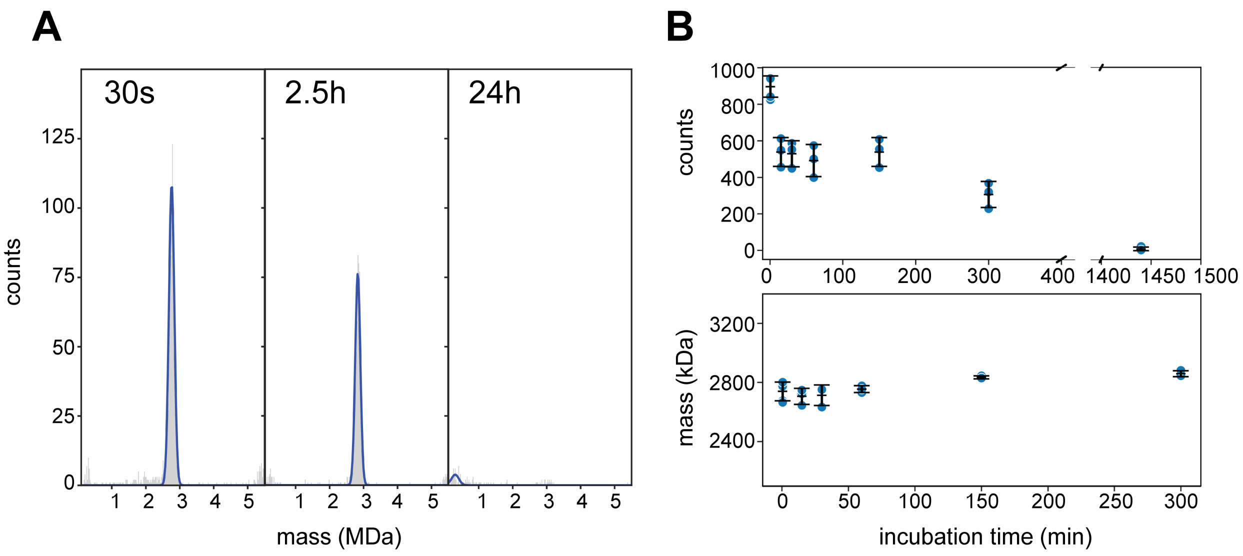

Figure S3: Stability of formic acid-treated pBR322. After treating pBR322 with 10% FA, the FA-treated samples were stored at 4 °C and measured at different timepoints. **A)** A few example acquisitions of the FA-treated pBR322 (storage times indicated at the top). **B)** Plotted over the different storage times are the counts (measured between 2 and 4 MDa) and the average mass of landing events of the ssDNA-like pBR322 particles. The depicted error bars represent the standard deviation. With some loss of signal within the first minutes after adding 10% FA, the ssDNA-like particles remain stable and can be measured up to several hours.

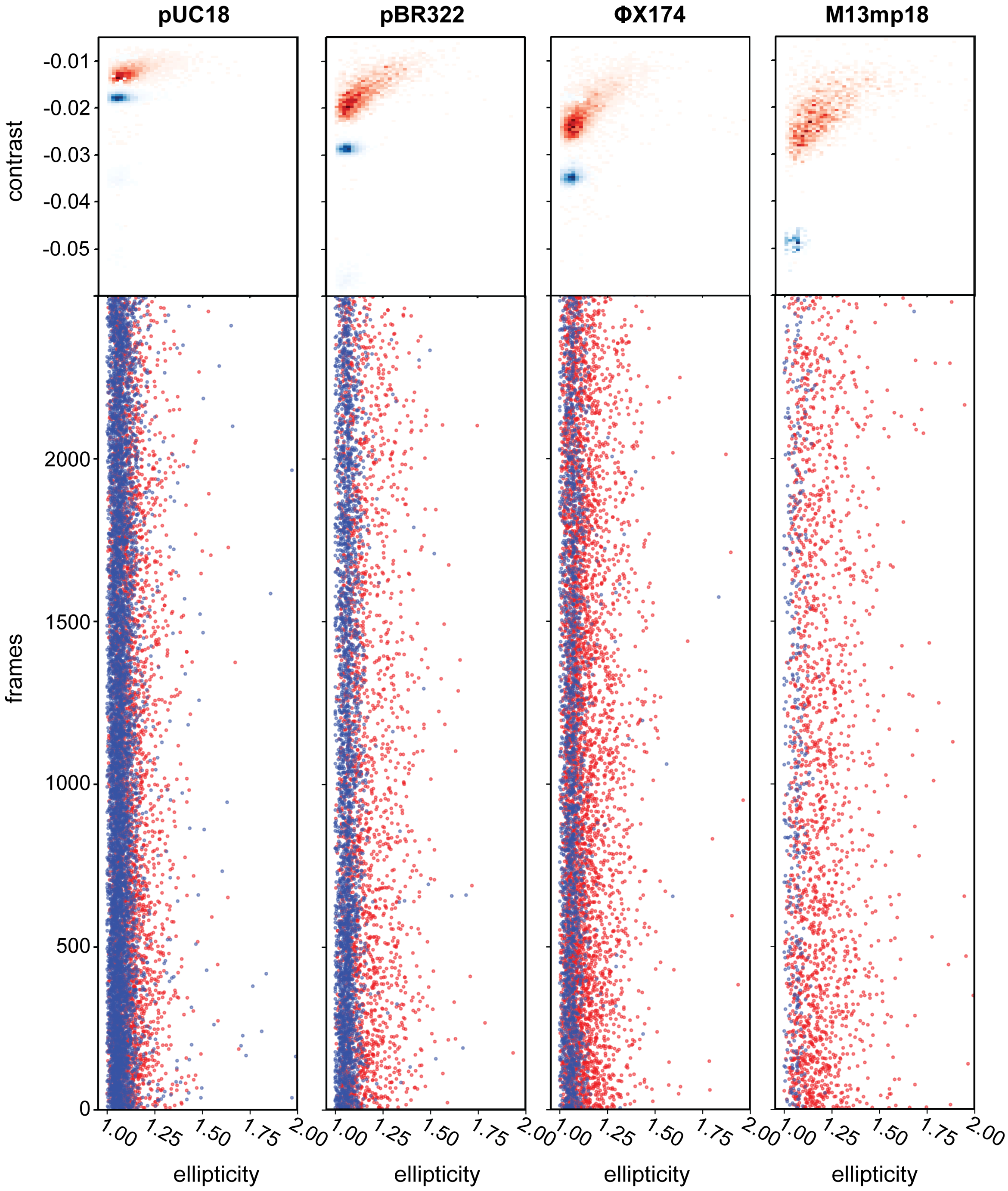

Figure S4: Characterization of non-ideal landing events for each of the studied pDNA constructs. Plotted in a 2D histogram are the contrast and ellipticity values of the landing events for the dsDNA plasmids on an APTES-coated glass slide. These landing events were monitored using the native particles (red) or after incubation in 10% formic acid (FA) for 30 s (blue). When not incubated with FA the pDNA particles yield non-circular landing events that impair correct mass determination. Plotting the ellipticity per measurement frame shows that the non-ideal landing events persist throughout the MP recording. Following short incubation with 10% FA the particles' landing events are mostly circular and show less spread in contrast values, enabling the correct mass analysis by MP.

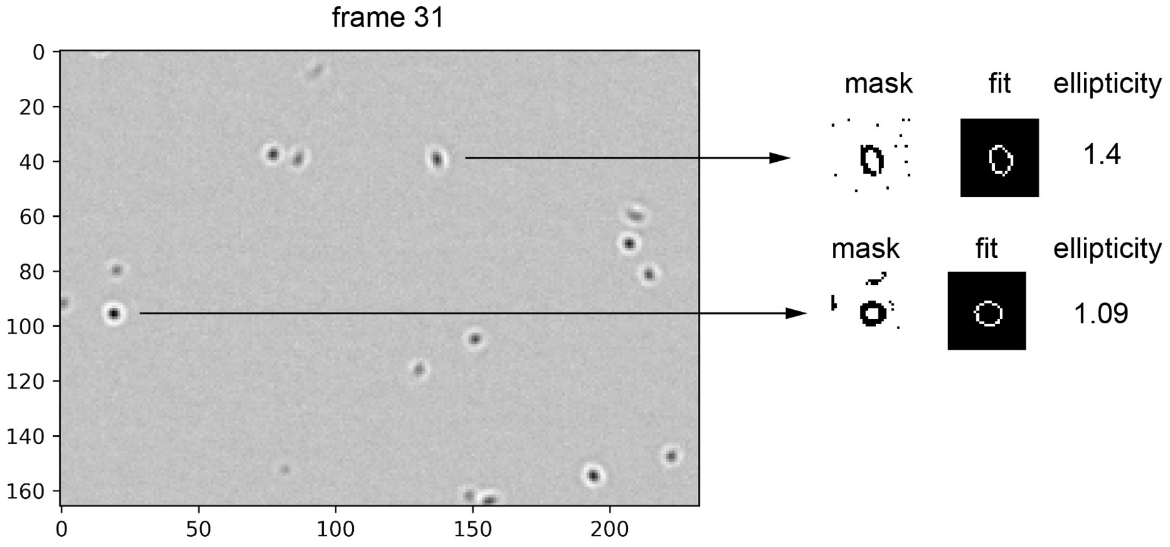

Figure S5: Illustrative example of the extraction of ellipticity values. Shown is a frame of a pBR322 recording from which two landing events are evaluated for their ellipticity. Following identification of the landing event in the frame, an individual mask is extracted isolating the typical interferometric Airy ring. After fitting, the ellipticity is calculated by dividing the width of the fitted ellipse by its height.
